# Nitrogen Application Timing and Levels Affect the Fate and Budget of Fertilizer Nitrogen in the Apple–Soil System

**DOI:** 10.3390/plants13060813

**Published:** 2024-03-12

**Authors:** Fen Wang, Chaoran Wang, Binghao Yang, Xinyu Luo, Gaowei Qi, Fajin Ji, Xinkai Guo, Tao Yang, Xuehui Zhao, Ming Li, Qianqian Jiang, Ling Peng, Hui Cao

**Affiliations:** 1 School of Advanced Agricultural Sciences, Weifang University, Weifang 261061, China; fenwang@wfu.edu.cn (F.W.); 19709656512@163.com (B.Y.); luowill1221@163.com (X.L.); 13676368263@163.com (G.Q.); 17306368595@163.com (F.J.); 18063274689@163.com (X.G.); 15253068797@163.com (T.Y.);; 2 Agriculture & Forestry Technology College, Weifang Vocational College, Weifang 261061, China; 3 Shandong Key Laboratory of Eco-Environmental Science for Yellow River Delta, Shandong University of Aeronautics, Binzhou 256600, China

**Keywords:** apple, ^15^N labeling, N fate, N use efficiency, microbial biomass N

## Abstract

This study aimed to determine the effects of the nitrogen (N) application period and level on the fate of fertilizer N and the contribution of N absorption and translocation to apple organ N. Two N application periods (labeled by the ^15^N tracer technique in spring and summer, represented by SP and SU, respectively) and three N levels (N0, MN, and HN) were used to determine the physiological indexes and aboveground, root, and soil ^15^N content of 4-year-old dwarf (‘Red Fuji’/M9T337) and arborized (‘Red Fuji’/*Malus hupehensis* Rehd.) apple trees. The results showed that HN led to shoot overgrowth, which was not conducive to the growth of the apple root system (root length, root tips, root surface area, and root volume) or the improvement of root activity. The contribution of soil N to apple organ N accounted for more than 50%, and the contribution of N application in summer to fruit N was higher than that in spring. Under HN treatment, the proportion of soil N absorbed by trees decreased, while that of fertilizer N increased; however, the highest proportion was still less than 50%, so apple trees were highly dependent on soil N. Under MN treatment, fertilizer N residue was similar to soil N consumption, and soil N fertility maintained a basic balance. Under HN treatment, fertilizer N residue was significantly higher than soil N consumption, indicating that excessive N application increased fertilizer N residue in the soil. Overall, the ^15^N utilization rate of arborized trees (17.33–22.38%) was higher than that of dwarf trees (12.89–16.91%). A total of 12.89–22.38% of fertilizer ^15^N was absorbed by trees, 30.37–35.41% of fertilizer ^15^N remained in the soil, and 44.65–54.46% of fertilizer ^15^N was lost. The ^15^N utilization rate and ^15^N residual rate of summer N application were higher than those of spring N application, and the ^15^N loss rate was lower than that of spring N application. High microbial biomass N (MBN) may be one of the reasons for the high N utilization rate and the low loss rate of N application in summer.

## 1. Introduction

China holds the top position globally in both apple cultivation area and apple yield [1]. Nitrogen (N) fertilizer plays a decisive role in increasing yield in the barren orchard soil of China. To ensure large fruit and high yield, the amount of N fertilizer used in the main apple-producing areas in China continues to increase, which far exceeds the demand of the trees [2,3]. Excessive unused N accumulates in the soil, which can easily cause deep leaching and surface runoff loss [4,5].

After N fertilizer is applied to the orchard, it is absorbed by the trees through the roots system to meet the needs of growth and development; however, it exists in the form of inorganic N or in combination with organic N (such as microbial biomass N (MBN)) in the soil profile. Other N is lost to the environment from the plant–soil system through processes such as ammonia volatilization, nitrification/denitrification, and leaching [6]. The fate of fertilizer N is influenced by crop type, soil type, N application rate, fertilization method, and fertilization period, which vary greatly [7,8]. Ge et al. [9] showed that the soil residual rate and loss rate of fertilizer N in 2-year-old apple orchards were 19.38–31.28% and 21.50–29.13%, respectively.

For the fertilization period, due to the high activity of N transformation (including ammonia volatilization, nitrification/denitrification, and leaching), separate fertilization generally has a higher N use efficiency than one-time fertilization. Researchers have found that the utilization rate of topdressing N is significantly higher than that of base fertilizer, while the loss rate is lower than that of base fertilizer [8,10,11]. Most studies only focus on N use efficiency and do not consider the residue of fertilizer N in the soil—that is, the compensation effect on the soil N pool. In the period of N application in apples, trees are fertilized in spring and summer [12]. Furthermore, the two fertilizer application periods corresponded to the two peak periods of apple root growth. However, differences in the direction of N fertilizer application and the absorption of apple trees in different periods have rarely been reported.

The ^15^N isotopic tracer technique has been used to monitor the budget of N fertilizer and is still widely used to evaluate fertilization management [13,14,15]. The ^15^N tracer method can quantify the source of N absorbed by the plant—that is, the amount of N from fertilizer and soil. If the amount of N from the soil is greater than the residual fertilizer N, it means that the residual fertilizer N cannot maintain the balance of the soil N pool. In contrast, residual fertilizer N can maintain the balance of the soil N pool. Therefore, the ^15^N tracer method can better reflect the compensation effect of fertilization on the soil N pool.

Therefore, the present study used ^15^N isotope tracing technology to determine the effects of N supply levels and N application periods (spring and summer) on N absorption, utilization, residue, and loss in apples. This can further clarify the relationship between fertilizer N, soil N, and tree N, improve N management in apple production, improve N application efficiency, and reduce negative impacts on the environment, which is of great significance for improving N conservation and efficiency in apple production.

## 2. Results

### 2.1. Biomass

**Figure 1:**
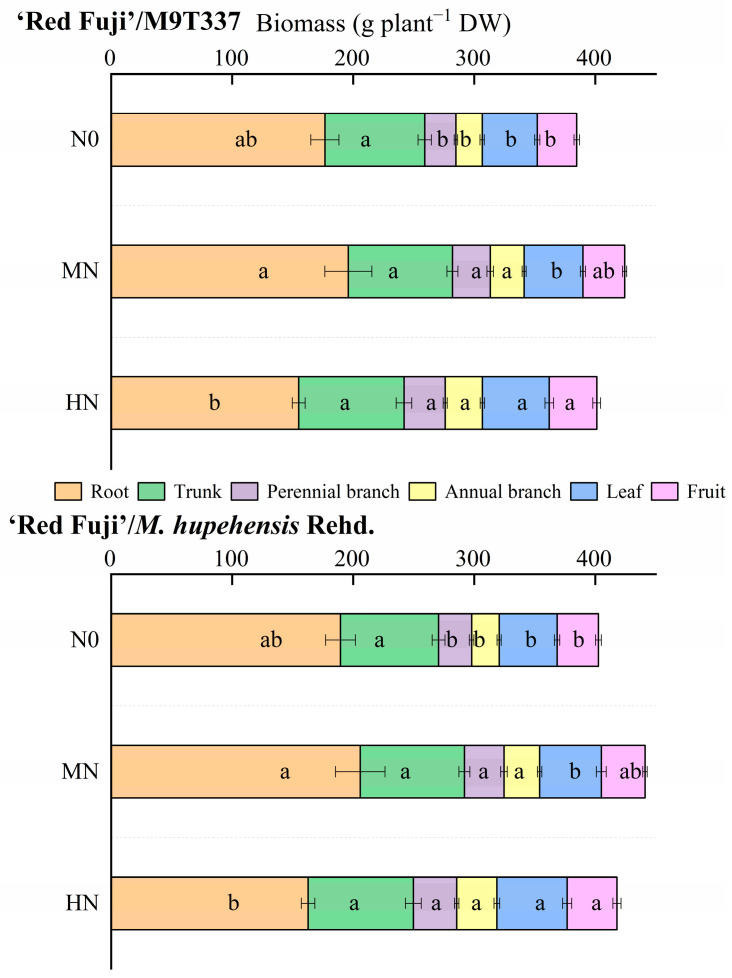
The biomass of apple organs. DW means dry weight. Error bar indicates the standard deviation of three replications. Different letters indicate statistically significant differences (*p* < 0.05).

Different levels of N application had different effects on the biomass of apple organs (Figure 1 and Table S1). Overall, the total biomass of arborized trees was higher than that of dwarf trees. With the increase in the N application level, the root biomass first increased and then decreased. The root biomass of the HN treatment was lowest, at 13.98% (‘Red Fuji’/M9T337) and 16.44% (‘Red Fuji’/*M. hupehensis* Rehd.) lower than the N0 treatment (*p* > 0.05). With the increase in the N application level, the biomass of annual branch, leaf, and fruit increased gradually and was highest under the HN treatment. HN was not conducive to root growth and led to shoot overgrowth.

### 2.2. Annual Branch Length, Leaf Area, and Chlorophyll Content

**Figure 2:**
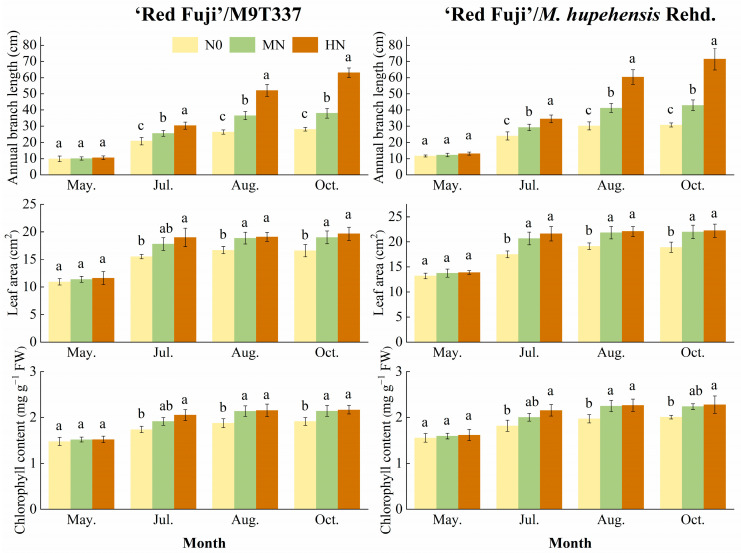
Annual branch length, leaf area, and chlorophyll content of apples. Error bar indicates the standard deviation of three replications. Different letters indicate statistically significant differences (*p* < 0.05).

Figure 2 and Table S2 show the effects of N application in spring and summer on annual branch length, leaf area, and chlorophyll content of ‘Red Fuji’/M9T337 and ‘Red Fuji’/*M. hupehensis* Rehd. under different N levels. Over time, the annual branch length, leaf area, and chlorophyll content of ‘Red Fuji’/M9T337 and ‘Red Fuji’/*M. hupehensis* Rehd. increased gradually. There was no significant difference in the new annual branch length in May under the N treatment, but it reached a significant level after July. In October, the growth rate of the new annual branch length of arborized trees was higher than that of dwarf trees. Compared with N0, MN and HN treatments increased the annual branch length and leaf area of ‘Red Fuji’/M9T337 and ‘Red Fuji’/*M. hupehensis* Rehd. to different degrees. In different periods, the chlorophyll content of the MN and HN treatments was higher than that of N0, although there was no significant difference in May. Over time, the chlorophyll content of ‘Red Fuji’/M9T337 and ‘Red Fuji’/*M. hupehensis* Rehd. showed an increase with the increase in N application level. HN had the most significant effect on annual branch length.

### 2.3. Root Activity and Root Morphology

**Figure 3:**
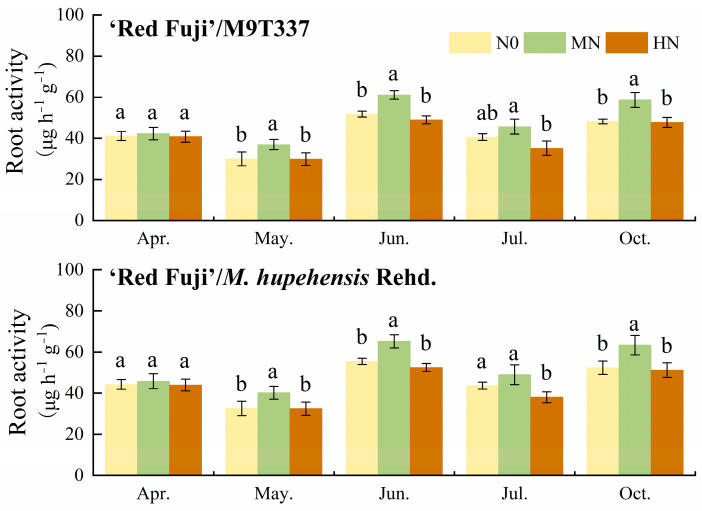
Root activity of apple under different N levels at different stages. Error bar indicates the standard deviation of three replications. Different letters indicate statistically significant differences (*p* < 0.05).

As shown in Figure 3 and Table S3, the effect of N application levels on apple root activity differed. Sampling in different periods showed that compared with N0, the root activity of ‘Red Fuji’/M9T337 under MN was the highest, while HN decreased the root activity to different degrees, although the difference was not significant in April. Early April, early June, and early October correspond to the three peak periods of apple root growth (spring, summer, and autumn). The root activity of ‘Red Fuji’/M9T337 in early June and early October was relatively high (47.73–65.20 μg h^−1^ g^−1^), followed by early April (40.81–45.78 μg h^−1^ g^−1^), while the root activity in May and July was relatively low (29.88–48.98 μg h^−1^ g^−1^). ‘Red Fuji’/*M. hupehensis* Rehd. followed the same trend.

**Figure 4:**
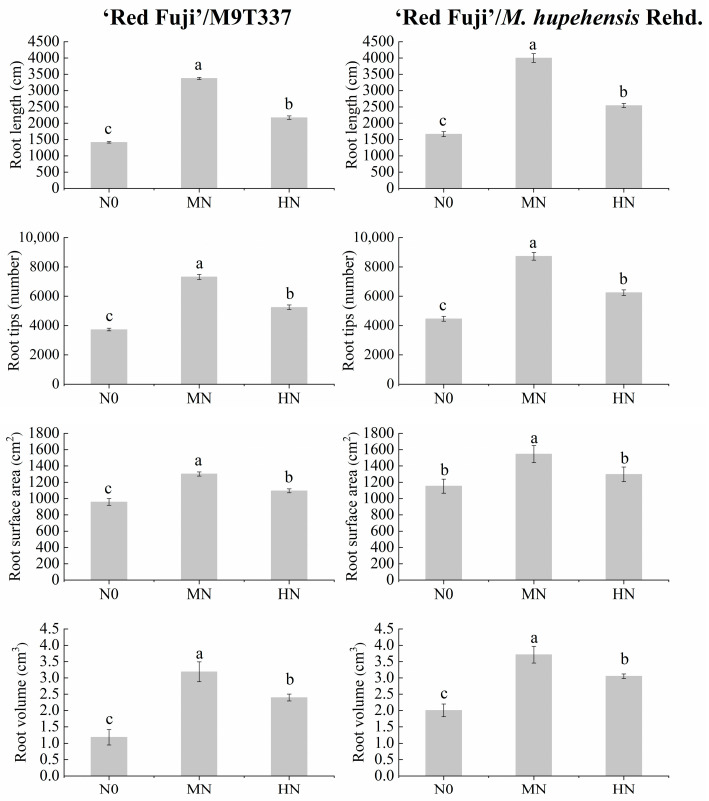
Root morphology of apples under different N levels during maturity stage. Error bar indicates the standard deviation of three replications. Different letters indicate statistically significant differences (*p* < 0.05).

N level had a significant effect on root morphology at the mature stage (Figure 4, Figure S1, and Table S3). With the increase in N level, the root length, root tips, root surface area, and root volume of ‘Red Fuji’/M9T337 increased first and then decreased, and were highest under the MN treatment. Compared with N0, the root length, root tip number, root surface area, and root volume of the MN treatment increased by 138.31, 95.95, 35.85, and 84.88%, respectively, and the differences all reached a significant level (*p* < 0.05). However, the root morphology of HN increased compared with N0 but decreased significantly compared with MN. ‘Red Fuji’/*M. hupehensis* Rehd. showed the same trend. The results showed that the HN treatment decreased root activity and was not conducive to root growth.

### 2.4. Apple Organ N Derived from Fertilizer (%Ndff) and Soil (%Ndfs)

**Figure 5:**
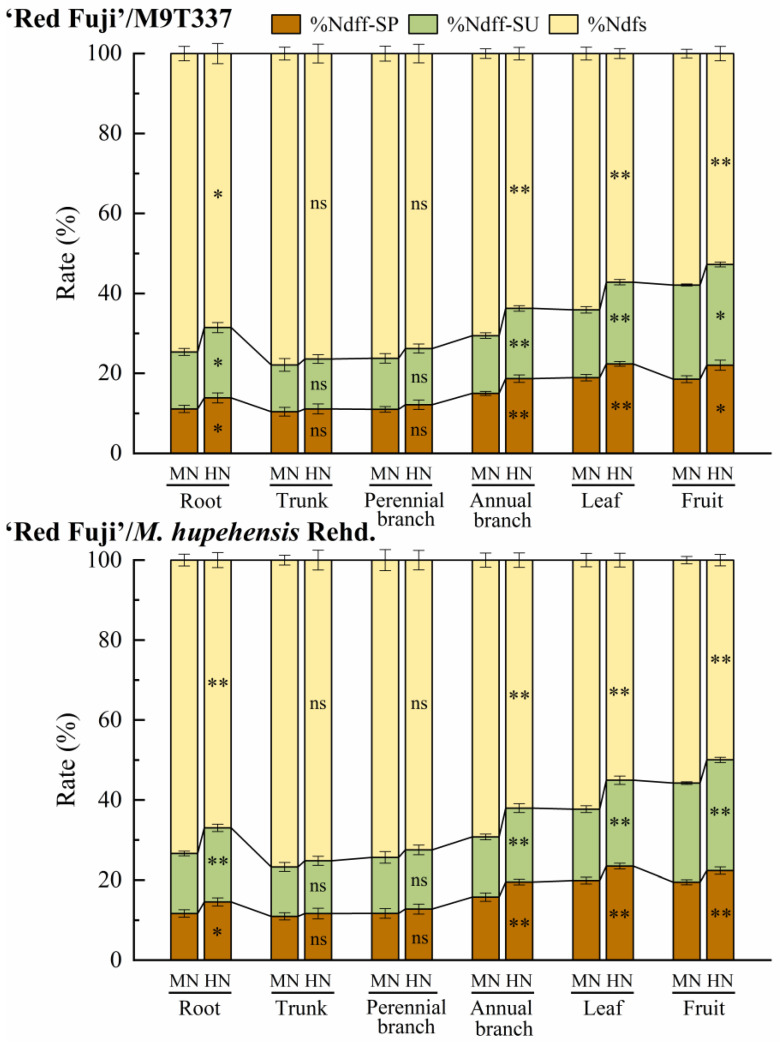
Contribution of spring N application (%Ndff-SP), summer N application (%Ndff-SU), and soil N (%Ndfs) to apple organ N. SP and SU represent N application in spring and summer, respectively. Error bar indicates the standard deviation of the three replications. * represents *p* < 0.05, ** represents *p* < 0.01, ns represents not significant.

The study of ‘Red Fuji’/M9T337 showed that the contribution of soil N to apple organ N (52.74–74.63%) was significantly higher than that of spring N application (10.43–22.39%) and summer N application (11.71–25.20%) (Figure 5 and Table S4). Under the MN treatment, fertilizer N contributed the most to fruit N (42.13%); spring N and summer N contributed 18.56% and 23.57%, respectively; fertilizer N contributed the least to trunk N (22.14%), and spring N and summer N contributed 10.43% and 11.71%, respectively. In contrast, soil N contributed the most to trunk N (77.86%) and the least to fruit N (57.87%). The HN treatment increased the contribution of spring and summer N applications and decreased the contribution of soil N to each organ. A comparison of different N application periods showed that a higher proportion of N applied in spring was transferred to annual branches (15.01–18.69%) and leaves (18.95–22.39%), while a higher proportion of N applied in summer was transferred to apple fruit (23.57–25.20%). ‘Red Fuji’/*M. hupehensis* Rehd. showed the same trend. The contribution of soil N to apple organ N was more than 50%, and the contribution of summer N application to fruit N was higher than that of spring N application.

### 2.5. ^15^N Residue

**Figure 6:**
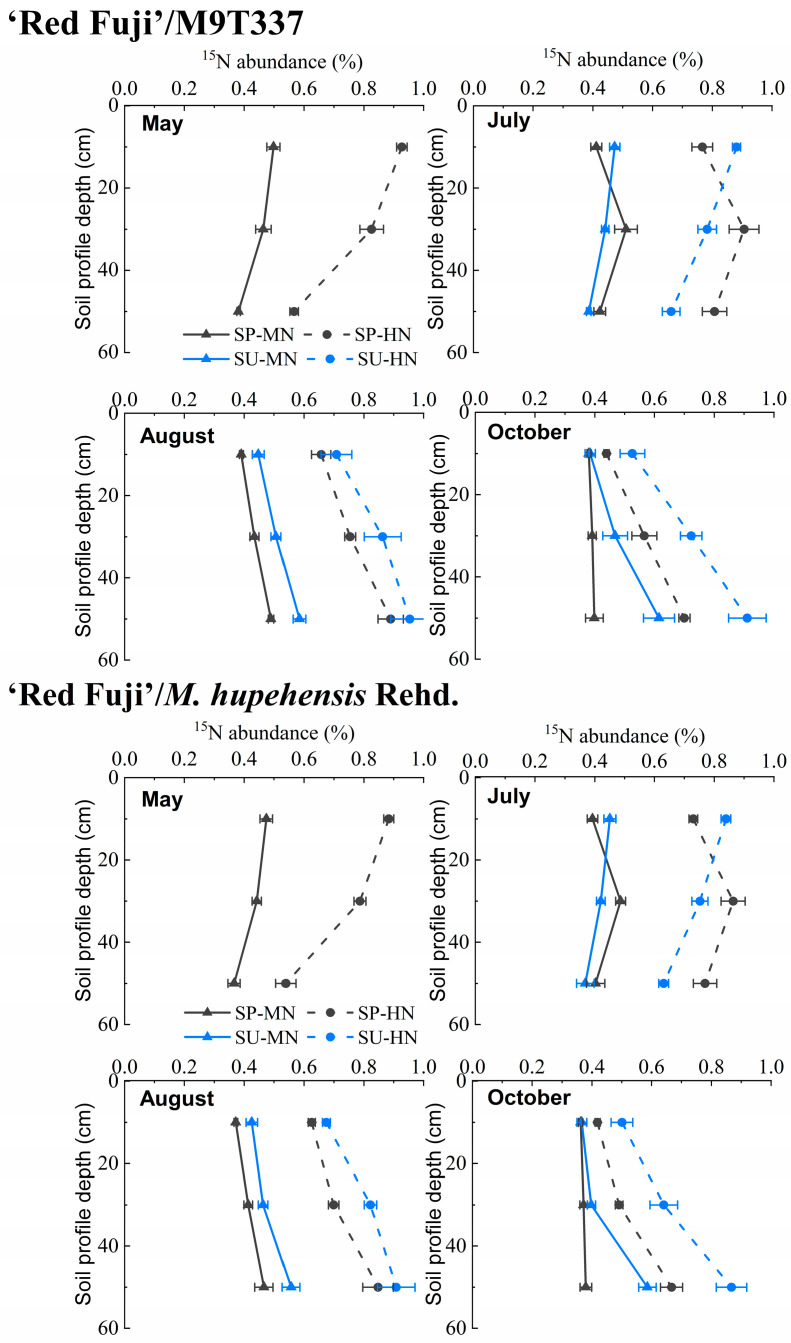
^15^N residue abundance in different soil layers under different treatments. SP and SU represent N application in spring and summer, respectively. Error bar indicates the standard deviation of the three replications.

Figure 6 shows the residual ^15^N abundance of soil in the root layer (0–60 cm) at different stages after N fertilizer application in the spring and summer. ‘Red Fuji’/M9T337 and ‘Red Fuji’/*M. hupehensis* Rehd. showed the same trend. In May (one month after applying N fertilizer in spring), the ^15^N abundance of the 0–40 cm soil layer was higher than that of the 40–60 cm soil layer; the abundance in each soil layer was higher under SP-HN treatment than under SP-MN. In July (one month after applying N fertilizer in summer), the ^15^N abundance of spring N application treatment (SP-MN and SP-HN) was highest in the 20–40 cm soil layer, which was significantly higher than that of the 0–20 cm and 40–60 cm soil layers. The trends in August and October were similar. With the deepening of the soil layer, the abundance of ^15^N increased gradually, and under the same amount of N application, the abundance of residual ^15^N in each soil layer was higher than that in spring. With the extension of time, the N application in spring and summer showed a downward trend, and the residue of N fertilizer applied in summer was higher than that in spring at the mature stage.

### 2.6. Utilization, Residue, and Loss of ^15^N

**Figure 7:**
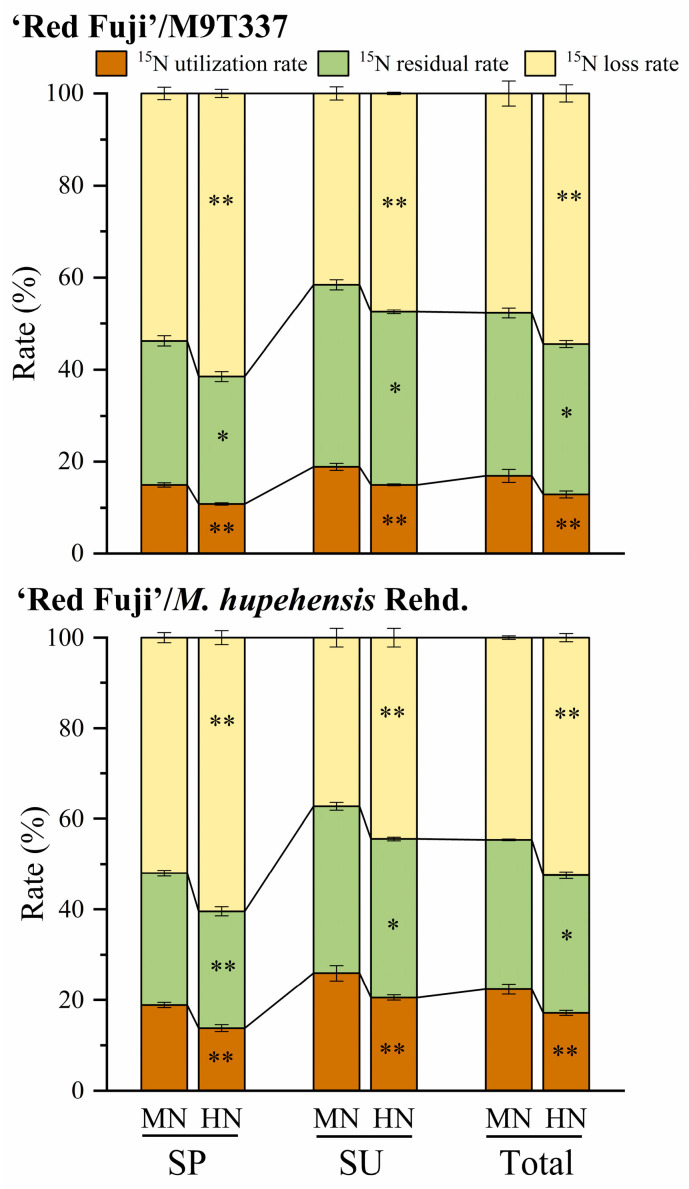
Utilization rate, residual rate, and loss rate of fertilizer ^15^N under different N application methods. SP and SU represent ^15^N application in spring and summer, respectively. Error bar indicates the standard deviation of the three replications. * represents *p* < 0.05, ** represents *p* < 0.01.

Figure 7 and Table S5 show the fate of fertilizer N from spring and summer N applications at different N levels. The study of ‘Red Fuji’/M9T337 showed that the ^15^N utilization rate of spring N application was lower than that of summer N application under MN and HN treatments. The total ^15^N utilization rate of the HN treatment was significantly lower than that of the MN treatment (decreased by 31.26%, *p* < 0.01), and the ^15^N utilization rate of spring N application and summer N application significantly decreased by 38.20 and 26.25%, respectively. Under the MN and HN treatments, the ^15^N residual rate of spring N application was lower than that of summer N application, and the ^15^N residual rate of the HN treatment was significantly lower than that of the MN (*p* < 0.05). The residual rates of spring and summer N application decreased by 12.91 and 5.17%, respectively. Under MN and HN treatments, the ^15^N loss rate of the spring N application was higher than that of the summer N application, and the ^15^N loss rate of the HN treatment was significantly higher than that of the MN treatment (increased by 14.24%, *p* < 0.01). The ^15^N loss rates of spring N application and summer N application increased by 14.33 and 14.13%, respectively. ‘Red Fuji’/*M. hupehensis* Rehd. also showed the same trend, and the ^15^N utilization rate (SP and SU) under different N levels was higher than that of ‘Red Fuji’/M9T337, while the residual rate and loss rate were lower than that of ‘Red Fuji’/M9T337. The ^15^N utilization rate and soil residual rate were higher and the loss rate was lower in summer, and the HN treatment significantly decreased the fertilizer ^15^N utilization rate and residual rate and increased the ^15^N loss rate.

**Figure 8:**
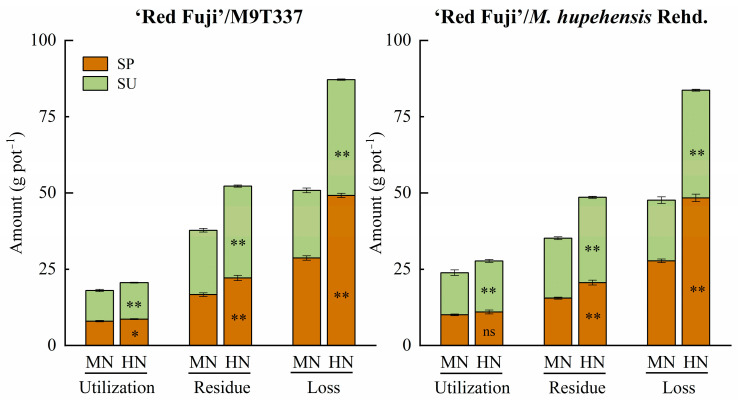
Utilization, residue, and loss of fertilizer N under different N application methods. SP and SU represent N application in spring and summer, respectively. Error bar indicates the standard deviation of the three replications. * represents *p* < 0.05, ** represents *p* < 0.01, ns represents not significant.

Figure 8 and Table S6 show the utilization, residue, and loss of fertilizer N under different N application levels and periods. Under MN and HN treatments, fertilizer N showed the following trend: loss > residual > absorption. The ^15^N uptake and residual amount of N applied in summer were higher than those applied in spring, and the ^15^N loss of N applied in spring was higher than that applied in summer. The HN treatment significantly increased the total absorption, total residue, and total loss of fertilizer N for ‘Red Fuji’/*M. hupehensis* Rehd. Residual fertilizer N in the soil was an important supplement to the consumption of the soil N pool. Under the MN treatment, the residual fertilizer N was similar to soil N consumption (Table 1), and soil N fertility maintained a basic balance. Under the HN treatment, the residual fertilizer N was significantly higher than soil N consumption, indicating that excessive N application increased the fertilizer N residue in the soil.

### 2.7. MBN

**Figure 9:**
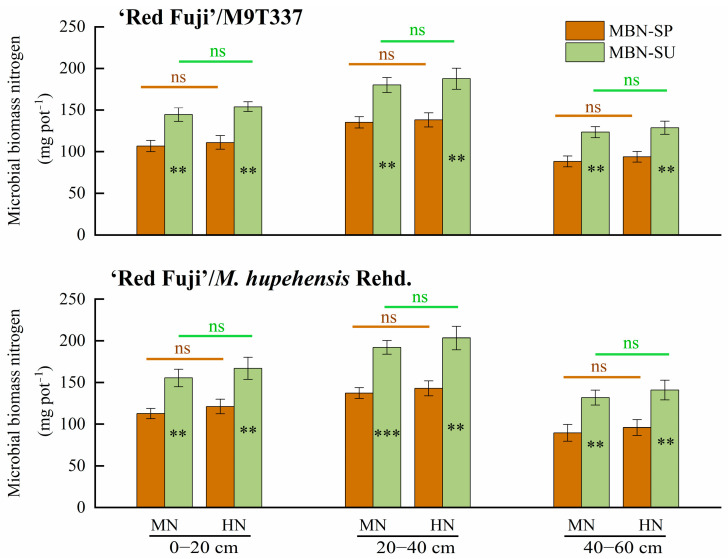
MBN-SP and MBN-SU contents in the 0–60 cm soil layer under different treatments. SP and SU represent N application in spring and summer, respectively. Error bar indicates the standard deviation of three replications. ** represents *p* < 0.01, *** represents *p* < 0.001, ns represents not significant.

Figure 9 and Table S7 show the MBN-SP and MBN-SU contents in the 0–60 cm soil layer under different treatments. Overall, the MBN content at 20–40 cm (135.15–203.40 mg pot^−1^) was higher than that at 0–20 cm (106.66–166.99 mg pot^−1^) and 40–60 cm (88.35–140.93 mg pot^−1^). The MBN content of HN in each soil layer was higher than that of MN, while the difference was not significant (*p* > 0.05). The MBN-SU content in each soil layer was significantly higher than that of MBN-SP (*p* < 0.01). Compared with MBN-SP, MBN-SU significantly increased by 35.46–38.74% (0–20 cm), 33.17–42.22% (20–40 cm), and 37.24–47.30% (40–60 cm), respectively.

## 3. Discussion

Higher root activity values indicate stronger root metabolic activities and nutrient absorption capacities [16]. Our results showed that root activity was inhibited under N0 and HN treatments, while the inhibitory effect was more obvious under HN than under N0 conditions. This is consistent with the study of Chen et al. [17] in pear seedlings.

The N absorbed by plants mainly comes from soil and fertilizer N. Stevens et al. [18] found that Ndfs accounted for 54–83% of the total N assimilated by maize, with the percentage decreasing with the long-term N application rate increasing, and explained that the increase in fertilization history reduced the absolute N uptake from soil sources. Yang et al. [11] showed that the N absorbed by summer maize mainly came from the soil, and the proportion of fertilizer N was less than 44%. In the present study, the contribution of soil N to apple organ N accounted for more than half. A comparison of different N application periods showed that a higher proportion of N applied in spring was transferred to annual branches and leaves, while a higher proportion of N applied in summer was transferred to apple fruit. Under the high N treatment, the proportion of soil N absorbed by trees decreased, while that of fertilizer N increased, but the highest proportion was still less than 50%, which shows that apple trees are highly dependent on soil N.

After applying N fertilizer into the soil, its utilization rate is 30–50%, and this varies greatly with different fertilization methods, soil properties, and management methods [19,20]. The timing and level of N application affect the N fate. The present study found that the N fertilizer utilization rate of spring N application ranged from 10.81 to 14.95%, while that of summer N application ranged from 14.96 to 18.88%. HN treatment significantly reduced the utilization and residual rate of fertilizer N and increased the N loss rate of fertilizer, consistent with Wang et al. [3]. This study also found that the ^15^N utilization rate of N application in summer was higher than that in spring due to strong root growth and more new roots in spring, but the growth period of new roots is relatively short. In summer, due to high temperatures, high humidity, a large leaf area in fruit trees, and high root vitality, root utilization efficiency after N application is higher. In addition, this study found that the N fertilizer utilization rate of arborized trees was higher than that of dwarf trees because the root morphology indicators (root length, root tips, root surface area, and root volume) and root activity of arborized trees were higher than those of dwarf trees. Thus, the root system of arborized trees has a higher absorption and utilization rate of fertilizer N. Webster [21] concluded that the decrease in absorption capacity was related to the smaller root systems in apple dwarf rootstocks and to the graft union, which had very convoluted xylem vessels that act as filters, affecting the balance of different solutes reaching its scion.

The portion of N not absorbed by the plants remains in the soil. Zhu [22] summarized that the residual rate of fertilizer N during crop harvests in China is generally 15–30%. The N residue rate of fertilizer in this experiment ranged from 25.78 to 39.56%. With the increase in the N application rate, the N residual amount increased, while the N residual rate decreased. This is consistent with Wang et al. [3]. The residual fertilizer N in the soil is an important supplement to the soil N pool. In this study, the ^15^N tracer method better reflected the compensation effect of N application in the soil N pool. Under MN treatment, fertilizer N residue was similar to soil N consumption, and soil N fertility maintained a basic balance. Under HN treatment, fertilizer N residue was significantly higher than soil N consumption, indicating that excessive N application increased fertilizer N residue in the soil. The abundance of ^15^N in the soil reflected the residual fertilizer N in each soil layer. The results showed that with the extension of time, both spring and summer N applications showed a downward trend of migration. The ^15^N residual abundance of summer N application in each soil layer was higher than that of spring N application, and the ^15^N residual abundance of high N treatment was higher than that of medium N treatment, indicating that excessive N application could aggravate the accumulation of fertilizer N in the soil.

MBN is closely related to soil N supply, which can be used as both an N source and N sink to regulate soil N supply through microbial immobilization and mineralization. Zhao et al. [23] showed that MBN was positively correlated with wheat biomass and N uptake, which could be used as an index of soil N supplying capacity. At the same time, microbial fixation of inorganic N was also an effective way to reduce N loss. Our research found that MBN-SP and MBN-SU were significantly correlated with N uptake by trees in SP and SU, respectively (*p* < 0.01), and significantly negatively correlated with N loss in SP and SU (*p* < 0.01). Therefore, high MBN-SU may be one of the reasons for high N use efficiency and low loss rate in SU. In this study, it was found that MBN content did not increase significantly under HN (Figure 9). The reason is that the application of high N decreases the C/N in soil, which is not conducive to the fixation of N by microorganisms. In addition, the limited carrying capacity of soil microorganisms under high N does not significantly increase MBN [24].

Under the conditions of continuous precipitation or massive irrigation, excessive nitrate N accumulates in the soil, which can be easily leached from the root zone to the deep soil layer, threatening the safety of shallow groundwater [25]. Other studies have shown that residual N in the soil is still available to future crops [11,26]. Therefore, in the late management of apple orchards, the water and fertilizer status should be reasonably regulated. The amount of N fertilizer should be reasonably controlled and the residual N resources in the root layer soil should be excavated to exert the aftereffect of residual N. However, it is necessary to reasonably regulate the water content of the root layer to prevent nitrate leaching [27].

The input of N fertilizer supplements the soil N pool, which is beneficial to improve soil fertility, but there are some problems, such as waste of resources and environmental pollution. Studies of winter wheat and summer maize in the North China Plain by Jia et al. [28] and Yang et al. [11] pointed out that when the amount of N fertilizer exceeded the crop demand, N loss increased sharply. Wang et al. [3] showed that the loss amount and rate of N fertilizer increased with an increase in the N application level. The same conclusion was drawn in the present study, and the trend was as follows: loss amount of N fertilizer > soil residual N > N absorbed by trees under medium and high N levels. Therefore, in fruit tree production, N fertilizer application should not only ensure the growth needs of trees and achieve efficient use of fertilizer N but also maintain the balance of the soil N pools and reduce N loss. This study showed that in the medium N treatment, the N utilization rate was higher and the N loss rate was lower. Under high N treatment, excessive N led to tree overgrowth, lower N utilization rate, and higher N loss rate, causing lower agricultural production efficiency and increasing the risk of environmental pollution.

## 4. Materials and Methods

### 4.1. Experimental Site and Materials

This experiment was performed from March 2022 to October 2022 at the Weifang University Modern Horticulture Research Institute located in Weifang, Shandong Province, China. The climate is semi-humid. The tested soil was collected from the surface layer of the apple orchard (0–20 cm) at the experimental station. Table S8 shows the average results of air-dried soil properties.

The test materials were 4-year-old apple trees. The variety was commercially important apple cultivar ‘Red Fuji’, which was grafted to dwarf rootstock M9T337 (‘Red Fuji’/M9T337) and arborized rootstock Malus hupehensis Rehd. (‘Red Fuji’/*M. hupehensis* Rehd.), respectively.

### 4.2. Experimental Design and Sampling

In an open space, pot experiments were carried out in pots with an inner diameter of 50 cm and height of 60 cm. The soil bulk density at the depth of 0−20, 20−40, and 40−60 cm was 1.09, 1.21, and 1.32 g cm^−3^, respectively, and the wet soil weight per pot was 142 kg. An apple tree was planted in each pot. The experiment was a two-factor design with 3 replicates. The application of N fertilizer in different periods was factor A, and there were two N fertilization periods: spring fertilization (SP) and summer fertilization (SU). The amount of N application was factor B, with no N application (N0), medium N application (MN, 0.21 g N kg^−1^ soil), and high N application (HN, 0.42 g N kg^−1^ soil) [29]. Of the total amount of N applied, 50% was applied in spring (March 20), and the remaining 50% was applied in summer (1 June). Each plant was treated with 0 (N0), 65 g (MN), or 130 g (HN) of urea. A total of 6.5 (MN)/13 (HN) g of normal urea applied per plant was replaced in each treatment with 6.5/13 g of ^15^N-urea (CO(^15^NH_2_)_2_, 10.22% abundance, Shanghai Research Institute of Chemical Industry) in spring or summer, namely ^15^N-SP+^14^N-SU and ^14^NH-SP+^15^N-SU, respectively. Calcium superphosphate (0.11 g kg^−1^ P_2_O_5_) and potassium sulfate (0.22 g kg^−1^ K_2_O) was applied as the base fertilizer to each plant. The fertilizers used and their composition percentages are shown in Table S9. The method of fertilization included digging a circular trench (radius of 30 cm, width and depth of 10 cm) around each tree. The field management measures such as diseases and insect pests in each growth period were consistent with the local conventional model. The soil moisture content was measured regularly using a soil moisture meter (Handi-TRASE), and when the soil moisture content was below the lower limit (60%), drip irrigation was performed.

**Figure 10:**
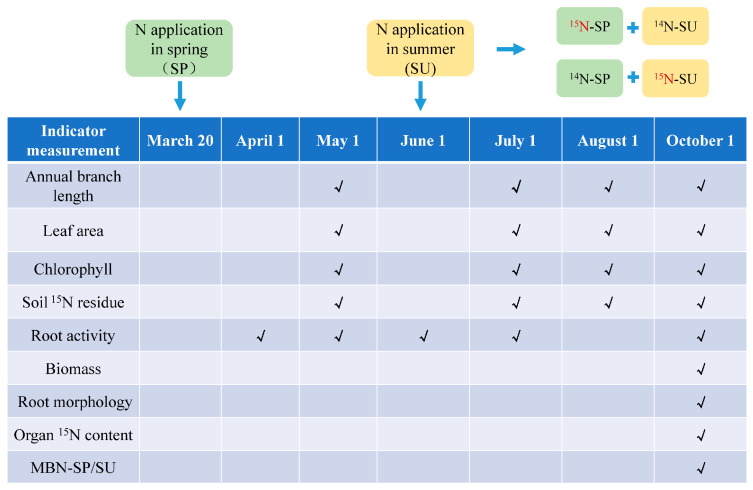
Processing and sampling time.

Twenty-four trees from two rootstocks were treated with each N level in each N application period. Among these, three trees of each rootstock were sampled and analyzed before treatment, and the N content of the plant was used as the basic value to calculate the N uptake of the trees. Annual branch length, leaf area, chlorophyll content, and soil ^15^N residues were measured in May, July, August, and October, respectively, and root activity was measured in April, May, June, July, and October (Figure 10).

Soil collection was carried out at 6 sampling sites where each pot was uniformly distributed. In the vertical direction of each sampling site, soil samples at depths of 0–20, 20–40, and 40–60 cm were collected, and 6 soil samples in each layer were evenly mixed into one replicate. After collection, the soil samples were transferred to the laboratory for ^15^N abundance determination. On October 1st, all plants were destructively sampled to determine the N content, ^15^N abundance, MBN, and root morphology.

### 4.3. Annual Branch Length, Leaf Area, and Chlorophyll Content

Ten annual branches and leaves were randomly selected from each tree to determine the annual branch length, leaf area, and chlorophyll content. The average of the 10 measured values for each tree was considered as one replicate, with a total of 3 replicates. The annual branch length was measured using a meter ruler. The leaf area was measured using a leaf area meter (YMJ-B, Zhejiang Top Yunong Technology Co., Ltd., Hangzhou, China). The chlorophyll concentration (total chlorophyll, chlorophyll a, and chlorophyll b) was calculated according to Li et al. [30].

### 4.4. Root Activity and Root Morphology

Root activity was measured using the triphenyltetrazolium chloride (TTC) method, and the reduction strength of tetrazolium (μg h^−1^ g^−1^ FW) was used to express root activity [31]. WinRhizo software (WinRHIZO Pro 2017a, Regent Instruments Canada) was used to analyze root morphological index [32].

### 4.5. ^15^N Abundance

Soil ^15^N-MBN was determined by chloroform fumigation extraction as described by Zhang et al. [13]. MBN was calculated as the difference in N content between fumigated and unfumigated samples divided by a conversion coefficient of 0.45 [13,33]. The ^15^N abundance of fumigated and unfumigated solution was determined as for inorganic ^15^N by DELTAV advantage isotope ratio mass spectrometer (Thermo Fisher Scientific Inc., Waltham, MA, USA) according to Zhang et al. [13].

Apple plants were divided into fruits, leaves, annual branches, perennial branches, trunk, and roots. The samples were heated at 105 °C for 30 min and dried at 80 °C, followed by homogenization and filtration with a 0.25 mm mesh screen. The N content was determined using the Kjeldahl method [34]. The ^15^N abundance of the plants and soil was determined using a DELTAV advantage isotope ratio mass spectrometer [6].

The calculation formulas are as follows:

(1) $$\%\mathrm{Ndff}=\frac{\mathrm{amount}\;\mathrm{of}\;\mathrm{fertilizer}{\;}^{15}N\;\mathrm{absorbed}\;\mathrm{by}\;\mathrm{trees}\;(g)}{\mathrm{amount}\;\mathrm{of}\;N\;\mathrm{absorbed}\;\mathrm{by}\;\mathrm{trees}\;(g)}\times 100\%$$

where %Ndff represents the percentage of N absorbed by tree from fertilizer.

%Ndfs = 100% − %Ndff(2)

where %Ndfs represents the percentage of N absorbed by tree from soil.

(3) $$\mathrm{Ndff}\;(\%)=\frac{\mathrm{abundance}\;\mathrm{of}{\;}^{15}N\;\mathrm{in}\;\mathrm{plant}\text{-}\mathrm{natural}\;\mathrm{abundance}\;\mathrm{of}{\;}^{15}N}{\mathrm{abundance}\;\mathrm{of}{\;}^{15}N\;\mathrm{in}\;\mathrm{fertilizer}\text{-}\mathrm{natural}\;\mathrm{abundance}\;\mathrm{of}{\;}^{15}N}\times 100\%$$

(4) $${\;}^{15}N\;\mathrm{utilization}\;\mathrm{rate}\;(\%)=\frac{\mathrm{Ndff}\times \mathrm{total}N\mathrm{of}\mathrm{organs}\;(g)}{{\;}^{15}N\;\mathrm{fertilization}\;(g)}\times 100\%$$

(5) $${\;}^{15}N\;\mathrm{residual}\;\mathrm{rate}\;(\%)=\frac{{\;}^{15}N\mathrm{residue}\;\mathrm{in}\;\mathrm{soil}\;(g)}{{\;}^{15}N\mathrm{fertilization}\;(g)}\times 100\%$$

^15^N loss rate (%) = 100% − ^15^N utilization rate (%) − ^15^N residual rate (%) (6)

### 4.6. Statistical Analysis

All graphs were plotted using Origin 8.0 (Northhampton, MA, USA). Data were analyzed with SPSS 20.0 (IBM Corporation, Armonk, NY, USA). The significance of differences between two and three treatments was analyzed using a *t*-test (Student’s *t*-test) and one-way factorial analysis of variance (ANOVA), respectively.

## 5. Conclusions

i: Soil N contributed more than half of the N of various apple organs, and N application in summer contributed more to fruit N than N application in spring.

ii: Under MN treatment, the fertilizer N residue was similar to soil N consumption, and soil N fertility maintained a basic balance. Under HN treatment, the fertilizer N residue was significantly higher than the soil N consumption, indicating that excessive N application intensifies the fertilizer N residue in the soil.

iii: The ^15^N utilization rate and ^15^N residual rate of summer N application were higher than those of spring N application, and the ^15^N loss rate was lower than that of spring N application. High MBN may be one of the reasons for high N utilization rate and low loss rate of N application in summer. The loss rate of N fertilizer was high (44.65–54.46%), and measures such as appropriately increasing the proportion of N application in summer, slow-release fertilizers, and water–fertilizer integration should be taken to reduce fertilizer N loss.

## Figures and Tables

**Table 1:** The amount of N absorbed by apple trees from fertilizer sources and soil sources.

Types of Trees	Treatment	Total N Uptake by Trees (g Plant^−1^)	N Absorbed from Fertilizer (g Plant^−1^)			N Absorbed from Soil (g Plant^−1^)
			Absorption of Spring N Application	Absorption of Summer N Application	Total	
‘Red Fuji’/M9T337	MN	55.03	7.97	10.07	18.04	36.99
	HN	64.15 ***	8.65 *	11.96 **	20.62 **	43.53 **
‘Red Fuji’/*M. hupehensis* Rehd.	MN	59.27	10.06	13.81	23.87	35.40
	HN	70.56 **	10.99 *	16.73 **	27.72 **	42.84 *

Note: * represents *p* < 0.05, ** represents *p* < 0.01, *** represents *p* < 0.001.

## Data Availability

All data are included in the main text and Supplementary Materials.

## Supplementary Materials

The following supporting information can be downloaded at https://www.mdpi.com/article/10.3390/plants13060813/s1, Figure S1: photos of dwarf rootstock M9T337 (‘Red Fuji’/M9T337) and arborized rootstock *Malus hupehensis* Rehd. (‘Red Fuji’/*M. hupehensis* Rehd.); Table S1: ANOVA analysis for the effects of studied factors (N rate and rootstock) on the biomass of apple organs. Significant (*p* < 0.05) values are highlighted using bold type; Table S2: ANOVA analysis for the effects of studied factors (N rate and rootstock) on annual branch length, leaf area, and chlorophyll content of apples. Significant (*p* < 0.05) values are highlighted using bold type; Table S3: ANOVA analysis for the effects of studied factors (N rate, DMPP application, and year) on root activity and root morphology. Significant (*p* < 0.05) values are highlighted using bold type; Table S4: ANOVA analysis for the effects of studied factors (N rate and rootstock) on %Ndff-SP, %Ndff-SU, and %Ndfs. SP and SU represent ^15^N application in spring and summer, respectively. Significant (*p* < 0.05) values are highlighted using bold type; Table S5: ANOVA analysis for the effects of studied factors (N rate and rootstock) on utilization rate, residual rate, and loss rate of fertilizer ^15^N. SP and SU represent ^15^N application in spring and summer, respectively. Significant (*p* < 0.05) values are highlighted using bold type; Table S6: ANOVA analysis for the effects of studied factors (N rate and rootstock) on utilization, residue, and loss of fertilizer N. SP and SU represent N application in spring and summer, respectively. Significant (*p* < 0.05) values are highlighted using bold type; Table S7: ANOVA analysis for the effects of studied factors (N rate and rootstock) on MBN-SP and MBN-SU. SP and SU represent ^15^N application in spring and summer, respectively. Significant (*p* < 0.05) values are highlighted using bold type; Table S8: chemical properties of experimental soil; Table S9: nutrient content of chemical fertilizer.
