# Supplementary material for: Nitrogen Application Timing and Levels Affect the Fate and Budget of Fertilizer Nitrogen in the Apple–Soil System
Source: Plants (Basel). 2024 Mar 12;13(6):813. doi: 10.3390/plants13060813 (PMC10975126; doi:10.3390/plants13060813)
Supplement: Supplementary file 1 [file plants-13-00813-s001.zip › plants-2878285-supplementary.pdf]

Nitrogen Application Timing and Levels Affect the Fate and Budget of Fertilizer Nitrogen in the Apple–Soil System

Fen Wang[1], Chaoran Wang[2], Binghao Yang[1], Xinyu Luo[1], Gaowei Qi[1], Fajin Ji[1], Xinkai Guo[1], Tao Yang[1], Xuehui Zhao[1], Ming Li[1], Qianqian Jiang[1], Ling Peng[3] and Hui Cao[1,*]

[1] School of Advanced Agricultural Sciences, Weifang University, Weifang, Shandong, China

[2] Weifang Vocational college, Weifang, 261061, Shandong, China

[3] Shandong Key Laboratory of Eco-Environmental Science for Yellow River Delta, Shandong University of Aeronautics, Binzhou, Shandong, China

* Correspondence: hui5232@163.com

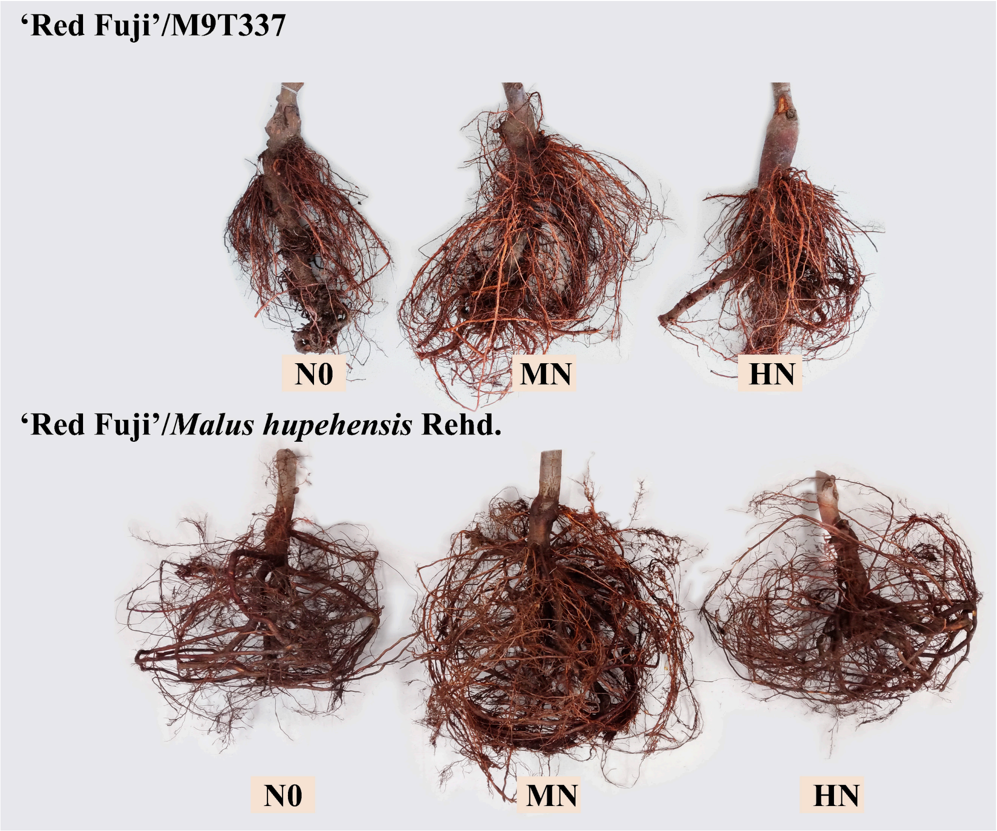

Figure S1. Photos of dwarf rootstock M9T337 (‘Red Fuji’/M9T337) and arborized rootstock *Malus hupehensis* Rehd. (‘Red Fuji’/*M. hupehensis* Rehd.)

Table S1. ANOVA analysis for the effects of studied factors (N rate and rootstock) on the biomass of apple organs. Significant (*P* < 0.05) values are highlighted using bold type.

Sources of variation	Root		Trunk		Perennial branch		Annual branch		Leaf		Fruit	
	F value	*P* value	F value	*P* value	F value	*P* value	F value	*P* value	F value	*P* value	F value	*P* value
N rate (N)	14.478	**<0.001**	1.894	0.185	25.052	**<0.001**	38.523	**<0.001**	16.342	**<0.001**	12.290	**<0.001**
Rootstock (R)	0.966	0.340	0.034	0.857	0.681	0.421	0.596	0.451	1.039	0.323	0.995	0.333
N × R	6.114	**<0.01**	0.630	0.681	10.265	**<0.001**	17.260	**<0.001**	7.079	**<0.01**	5.099	**<0.01**

Table S2. ANOVA analysis for the effects of studied factors (N rate and rootstock) on annual branch length, leaf area, and chlorophyll content of apples. Significant (*P* < 0.05) values are highlighted using bold type.

Sources of variation	Annual branch length		Leaf area		Chlorophyll content	
	F value	*P* value	F value	*P* value	F value	*P* value
N rate (N)	9.586	**<0.001**	3.699	**<0.05**	5.181	**<0.01**
Rootstock (R)	69.647	**<0.001**	10.178	**<0.01**	2.130	0.15
N × R	30.265	**<0.001**	1.448	**<0.001**	2.433	**<0.01**

Table S3. ANOVA analysis for the effects of studied factors (N rate, DMPP application and year) on root activity and root morphology. Significant (P < 0.05) values are highlighted using bold type.

Sources of variation	Root activity		Root length		Root tips		Root surface		Root volume	
	F value	*P* value	F value	*P* value	F value	*P* value	F value	*P* value	F value	*P* value
N rate (N)	7.863	**<0.001**	110.884	**<0.001**	61.494	**<0.001**	11.713	**<0.001**	30.260	**<0.001**
Rootstock (R)	2.807	0.097	0.874	0.364	1.666	0.215	6.876	**<0.05**	2.887	0.109
N × R	3.794	**<0.01**	557.057	**<0.001**	312.866	**<0.001**	25.344	**<0.001**	56.584	**<0.001**

Table S4. ANOVA analysis for the effects of studied factors (N rate and rootstock) on %Ndff-SP, %Ndff-SU and %Ndfs. SP and SU represent ^{15}N application in spring and summer, respectively. Significant ($P < 0.05$) values are highlighted using bold type.

Sources of variation	%Ndff-SP		%Ndff-SU		%Ndfs	
	F value	*P* value	F value	*P* value	F value	*P* value
N rate (N)	6.180	**<0.05**	5.351	**<0.05**	6.244	**<0.05**
Rootstock (R)	0.456	0.502	0.829	0.366	0.679	0.413
N × R	2.176	0.099	2.049	0.115	2.284	0.087

Table S5. ANOVA analysis for the effects of studied factors (N rate and rootstock) on utilization rate, residual rate, and loss rate of fertilizer ^{15}N. SP and SU represent ^{15}N application in spring and summer, respectively. Significant (P < 0.05) values are highlighted using bold type.

Sources of variation	^{15}N utilization rate-SP		^{15}N utilization rate-SU		^{15}N residual rate-SP		^{15}N residual rate-SU		^{15}N loss rate-SP		^{15}N loss rate-SU	
	F value	*P* value	F value	*P* value	F value	*P* value	F value	*P* value	F value	*P* value	F value	*P* value
N rate (N)	17.678	**<0.01**	4.618	0.057	25.947	**<0.001**	52.699	**<0.001**	105.663	**<0.001**	21.393	**<0.001**
Rootstock (R)	5.361	**<0.05**	20.309	**<0.001**	3.342	0.097	14.012	**<0.01**	0.275	0.612	3.185	0.105
N × R	524.419	**<0.001**	275.970	**<0.001**	97.002	**<0.001**	19.009	**<0.001**	43.460	**<0.001**	33.937	**<0.001**

Table S6. ANOVA analysis for the effects of studied factors (N rate and rootstock) on utilization, residue, and loss of fertilizer N. SP and SU represent N application in spring and summer, respectively. Significant (*P* < 0.05) values are highlighted using bold type.

Sources of variation	Utilization-SP		Utilization-SU		Residue-SP		Residue-SU		Loss-SP		Loss-SU	
	F value	*P* value	F value	*P* value	F value	*P* value	F value	*P* value	F value	*P* value	F value	*P* value
N rate (N)	1.311	0.279	3.116	0.108	136.184	**<0.001**	194.717	**<0.001**	3932.190	**<0.001**	333.302	**<0.001**
Rootstock (R)	65.453	**<0.001**	28.803	**<0.001**	0.636	0.444	0.427	0.528	0.018	0.896	0.248	0.629
N × R	191.449	**<0.001**	280.645	**<0.001**	356.014	**<0.001**	861.678	**<0.001**	3644.276	**<0.001**	548.569	**<0.001**

Table S7. ANOVA analysis for the effects of studied factors (N rate and rootstock) on MBN-SP and MBN-SU. SP and SU represent ^{15}N application in spring and summer, respectively. Significant (*P* < 0.05) values are highlighted using bold type.

Sources of variation	MBN-SP		MBN-SU	
	F value	*P* value	F value	*P* value
N rate (N)	14.701	**<0.01**	19.952	**<0.001**
Rootstock (R)	0.398	0.542	1.039	0.332
N × R	4.646	**<0.05**	8.943	**<0.01**

Table S8. Chemical properties of experimental soil.

Soil parameters	
pH (1:2.5 water extract)	7.87
EC (1:5 water exact; dS m^{-1})	0.65
Organic matter (Walkley-Black; g kg^{-1})	18.23
Alkali-hydrolyzed N (Alkaline hydrolysis diffusion method; g kg^{-1})	0.07
Available phosphorus (Olsen; sodium bicarbonate extract; g kg^{-1})	0.04
Available potassium (Ammonium acetate extract; g kg^{-1})	0.22
Exchangeable calcium (Ammonium acetate extract; g kg^{-})	0.49
Exchangeable magnesium (Ammonium acetate extract; g kg^{-1})	0.08

Note: Soil parameters are all based on soil dry weight.

Table S9. Nutrient content of chemical fertilizer.

Fertilizers	Nutrients
Urea	46% ^{14}N
^{15}N-urea	46% ^{15}N
Calcium superphosphate	14% P_2O_5, 13% Ca, 8% S
Potassium sulfate	50% K_2O, 18% S
